# Association of lifestyle habits and cardiovascular risk among sedentary adults

**DOI:** 10.1097/MD.0000000000034376

**Published:** 2023-07-21

**Authors:** Linyu Peng, Lidan Chen, Shen Wang, Lianmeng Guo, Wenhao Liang, Jie Zhou, Niujin Shi, Junhao Huang, Min Hu, Jingwen Liao

**Affiliations:** a Guangdong Provincial Key Laboratory of Physical Activity and Health Promotion, Guangzhou Sport University, Guangzhou, China; b Graduate School, Guangzhou Sport University, Guangzhou, China; c Scientific Research Center, Guangzhou Sport University, Guangzhou, China; d School of Foreign Languages, Neusoft Institute Guangdong, Guangzhou, China; e School of Sport and Health, Guangzhou Sport University, Guangzhou, China.

**Keywords:** arterial stiffness, autonomic nervous system function, cardiovascular risk, lifestyle habits, sedentary behavior

## Abstract

This study aimed to analyze the association of lifestyle habits (physical activity, sleep habits, and eating habits) with cardiovascular risk (arterial stiffness and autonomic nervous system function) among sedentary adults. Sixty adults of sedentariness and physical activity were evaluated by accelerometers; sleep and eating habits were assessed by questionnaires; cardiovascular risks were assessed by pulse wave velocity (PWV), ankle-brachial index, flow mediated dilation, and heart rate variability; circulating biomarkers were also determined. Prolonged sitting (represented by longer maximum length of sedentary bouts, lower length of sedentary breaks, and more total time of sitting) were (*P* < .05) significantly associated with matrix metalloproteinases, neuropeptide Y, C-reactive protein, peptide Y, ghrelin, and leptin; significant associations (*P* < .05) were also observed of total time in physical activity with most circulating biomarkers except interleukin-6, tumor necrosis factor-α, and adiponectin. Sleep habits, especially sleep efficiency, were (*P* < .05) significantly associated with PWV, ankle-brachial index, and circulating biomarkers. Eating habits (including emotional overeating and enjoyment of food) were (*P* < .05) significantly associated with PWVs and flow mediated dilation; satiety responsiveness and enjoyment of food were (*P* < .05) significantly associated with low-frequency spectral component expressed in normalized units, high frequency spectral component expressed in normalized units, and ratio between low-frequency/high frequency spectral component expressed in normalized units. The findings indicated that several lifestyle habits among sedentary adults were closely associated with increased cardiovascular risk. Sedentary people were encouraged to live with sufficient physical activity, good sleep, and healthy eating habits for decreasing arterial stiffness and balancing autonomic nervous function.

## 1. Introduction

Sedentary behaviors include any behavior characterized by an energy expenditure of < 1.5 metabolic equivalent task in seated, reclined or lying postures.^[1]^ Nowadays, sedentary behaviors have become one of the most common behavior patterns in modern society^[2]^; adults spend about 1/3 to 1/2 of daily time in sedentary behavior,^[3]^ such as sitting work, leisure-time activities before screens, and even taking public transports. Moreover, sedentary adults have also been recognized to live with other unhealthy lifestyle habits including poor sleep habits,^[4]^ unbalanced eating habits,^[5]^ and a lack of physical activity.^[6]^ Existing literatures demonstrated that prolonged sedentary adults tends to have an elevated risk of insomnia and sleep disturbance,^[7]^ which is simultaneously accompanied by autonomic nervous system dysfunction and impairment of vascular function.^[8,9]^ In addition, sedentary behaviors have also been shown to interact with dietary habits, such as over consumption of unhealthy food and excessive calorie intake,^[1]^ which consequently lead to overweight, obesity, insulin resistance, and therefore increased cardiovascular risks.^[10]^ Moreover, physical active and prolonged sitting are now studies as distinct but interrelated behavioral attributes; available evidences emphasize that a combination of low physical activity and prolonged sitting would augment cardiovascular risk.^[11,12]^

Those unhealthy lifestyle habits among sedentary adults would interact with each other^[13,14]^ and produce alarming consequences related to cardiovascular complications.^[15]^ Every 1 hour increased of sedentary behavior was seen to be associated with 11% higher risk of all-cause mortality and 18% greater risk of cardiovascular risk mortality.^[16]^ Long-term sedentary behavior, as an unhealthy lifestyle, would lead to impairment in vascular function and arterial wall stiffening.^[17]^ Arterial stiffness is a predictor of cardiovascular morbidity and mortality in various chronic disease among sedentary populations.^[18]^ Given the predictive power of pulse wave velocity (PWV), flow mediated dilation (FMD) and ankle-brachial index (ABI), identifying strategies that prevent or reduce stiffening may be important in the prevention of cardiovascular events. Additionally, sedentary lifestyle negatively alters autonomic nervous system activity as reflected by decreased heart rate variability (HRV), and thus increases cardiovascular risks.^[19]^ HRV provides a simple measure to illustrate the shift of autonomic nervous system function and thus is capable of predicting cardiovascular risk. In addition to cardiovascular dysfunction commonly observed among those with unhealthy lifestyle habits, circulating biomarkers indicating subclinical changes are also necessary; sedentary behavior has been also linked to low-grade inflammation, adipokines, and appetite-regulating hormones, independently of physical activity levels.^[20]^

Available evidence has linked sedentary behaviors with an increased risk of cardiovascular disease. Moreover, sedentary populations have also been recognized to live with other unhealthy lifestyle habits including lack of physical activity, poor sleep habits, and unhealthy eating habits. However, the association of those lifestyle habits with cardiovascular risks among sedentary population has not been fully demonstrated, especially through comprehensive measures based on both subjective and objective methods. Our study would provide a better understanding of cardiovascular risk among sedentary adults.

## 2. Materials and methods

### 2.1. Study design and participants recruitment

**Figure 1.**
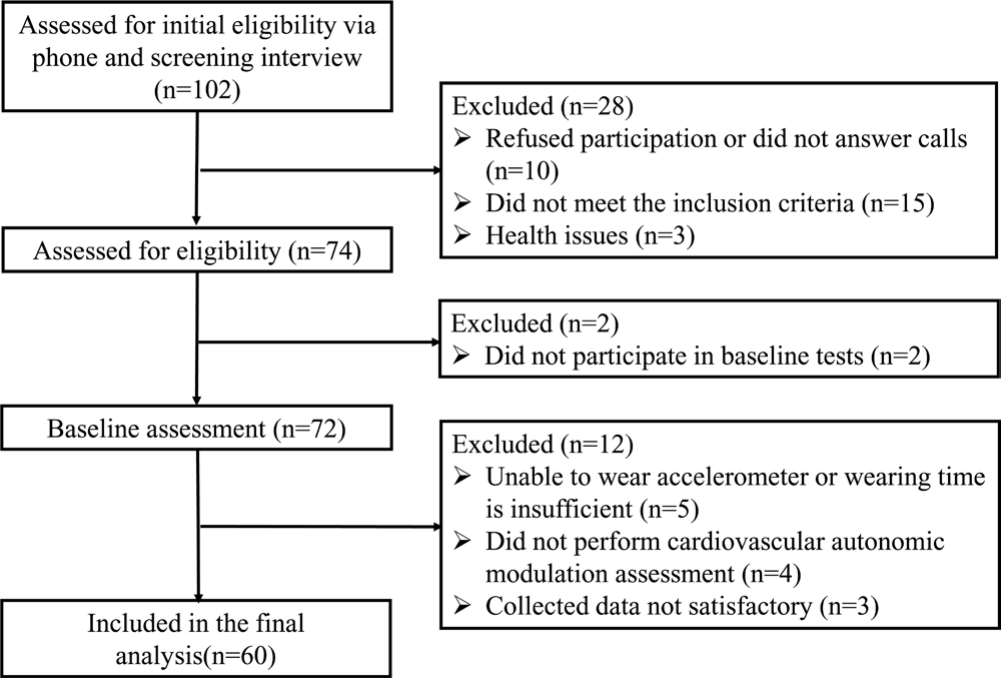
This cross-sectional study (refer to Figure 1) was conducted at the guangdong provincial key laboratory of physical activity and health promotion.

This cross-sectional study (refer to Fig. 1) was conducted at the Guangdong Provincial Key Laboratory of Physical Activity and Health Promotion. Eligible participants met the following criteria: Aged 18 to 35 years; Sedentary time > 8 hours; and Not to suffer from any cardiovascular risk. The following exclusion criteria were used: Use of medications that could interfere with the studied variables; Osteoarticular problems that would impair the practice of physical exercise; and Cardiovascular and metabolic diseases.

This study was approved by the Human Experimental Ethics of the Guangzhou Sport University (ID Number: 2021LCLL-06). All included participants provided informed consent forms. Demographic data (name and age), anthropometric data, lifestyle habits (sedentary behaviors, physical activity, sleeping habits, and eating habits), and cardiovascular data were collected by interviewing or evaluating between 7:00 AM and 11:50 AM at the laboratory The participants were instructed fasting, enough sleep, not to engage in vigorous physical activity, and not to drink alcoholic beverages for the 12 hours prior to the evaluations.

### 2.2. Anthropometric data collection

Height was determined with participants wearing light clothing and without shoes using an altimeter (model SH-8053, China). Subsequently, body composition was measured by Bio-eletrical Impedance (InBody 370, Korea) strictly following the manufacturer’s instructions, obtaining fat mass, waist–hip ratio, body mass index.

### 2.3. Evaluation of lifestyle habits

#### 2.3.1. Evaluation of sedentary behaviors and physical activity using accelerometer.

Sedentary time and physical activity time were measured by using accelerometer (ActiGraph GT3X + model, USA). Accelerometer was used for 7 consecutive days, being affixed to the nondominant wrist. Volunteers were informed not to remove the device for sleeping, bathing, as the equipment is small and waterproof. The data collected were downloaded using ActiLife 6 software (ActiGraph, USA). The devices were activated on the first day in the morning and data were recorded using the raw mode with a 30 Hz frequency, and posteriorly downloaded into 60-second epochs. Apart from accelerometer non-wear time, a valid day was defined as has 10 hours or more of monitor wear, all volunteers with at least 5 valid days were included in the analyses. A sedentary bout is defined as a minimum length of 10 consecutive minutes of sedentary time, a break in sedentary time was defined as all interruptions (lasting at least 1-minute) in sedentary time when the recorded counts value was > 100 cpm. Each minute during which the accelerometer counts were below 100 cpm was defined as sedentary time. Accelerometer counts ≥ 100 cpm were classified as physical activity with additional separation into light-intensity (101-1951 cpm); moderate-intensity (1952-5724 cpm) and vigorous physical activity (≥5725 cpm). Using the ActiLife software, we obtained the following sedentary behavior and physical activity variables: Maximum length of sedentary bouts; Total length of sedentary breaks; Total time in sedentary behavior; Moderate to vigorous physical activity time; and Total time in physical activity.

#### 2.3.2. Assessment of sleep habits using questionnaire and accelerometer.

Sleep quality was indicated by the Pittsburgh sleep quality index and accelerometer data. The questionnaire included 7 aspects: Subjective sleep quality; Sleep latency; Sleep duration; Habitual sleep efficiency; Step disturbances; Use of sleeping medication; Daytime dysfunction, each of which has a range of 0 to 3 points. The maximum score of the sleep quality index is 21 points, and scores higher than 5 indicate poor sleep quality^[21]^; with higher scores indicating poorer sleep quality.

Accelerometer data would be collected and evaluated from the 7 nights, subject sleep time were entered into the ActLife software for sleep analysis, with validated algorithms developed to determine walking vs. sleep movement, the following indices were obtained: sleep efficiency [(total sleep time/time in bed) *100], average total sleep time (total sleep time in minutes/7), and wake after sleep onset (number of minutes of wake time after defined sleep onset). Poor sleep was defined as a night of total sleep time < 6 hours or sleep efficiency < 85%.

#### 2.3.3. Determination of eating behaviors through questionnaire.

Eating habits were assessed using the Adult Eating Behavior Questionnaire developed by Hunot et al.^[22]^ The questionnaire is a 35-item with 5-point Likert scale, which assess the eating habits through the near month. The questionnaire described appetitive traits, including food approach and food avoidance traits,^[23]^ and focused on 8 aspects: Food responsiveness; Emotional overeating; Enjoyment of food; Satiety responsiveness; Emotional under-eating; Food fussiness; Slowness in eating. The scores for each item are based on the Likert scale, and ratings are attributed to specific scores – from 1 point (“strongly disagree”) to 5 points (“strongly agree”), while some items are defined as reverse ones – from 1 point (“strongly agree”) to 5 points (“strongly disagree”); with higher scores indicating worse eating habits (represented by emotional overeating and emotional under-eating) or better dietary (represented by food responsiveness, enjoyment of food, satiety responsiveness, food fussiness and slowness in eating).

### 2.4. Evaluation of cardiovascular risk

#### 2.4.1. PWV analysis.

Arterial stiffness was quantified by carotid-femoral pulse wave velocity (Cfpwv) using the SphygmoCor device (AtCor Medical, Australia). Measurement of brachial-ankle pulse wave velocity (Bapwv) was carried out as described in previous studies.^[24]^ Cfpwv is broadly recognized as the gold-standard measurement of arterial stiffness.^[18,25]^ In order to control for the effects of food and physical activity on Cfpwv, we instructed participants to refrain from food, caffeinated beverages, tobacco, medications, or vigorous physical activity within 8 hours before the assessment. All measurements were performed with the subjects in a supine position in a quiet room. Participants were first asked to rest in a supine position for 10 minutes. Next, we used a measuring tape to measure the body surface distance from the suprasternal notch to the right common carotid artery site (s-carotid distance) and the body surface distance from the suprasternal notch to the right common femoral artery site (s-femoral distance). A pressure-sensitive tonometer was then lightly pressed over the carotid and femoral pulse points, sequentially, to acquire a minimum of 12-second pulse wave signals referenced to electrocardiogram at each arterial site.^[26]^ To maintain consistency, all assessments were carried out by 1 trained researcher.

#### 2.4.2. ABI analysis.

The ABI was determined using the oscillometric method with a Boso ABI-System 100 device (BOSCH + SOHN GmbH u. Co. KG, Germany) which enables simultaneous measurement of systolic blood pressure on all 4 extremities. All volunteers were familiarized with the laboratory procedures, and they remained in supine position over 15 minutes monitoring duration, thus ensuring that they remained completely relaxed during the recording data. Fluctuations in the individual measurement duration are reduced to a minimum through the simultaneous pump system and regulation of release speed. After measuring, the values are transmitted to a computer, where the application software automatically calculates the ABI. The diagnosis of peripheral arterial disease was based on an ABI value of <0.9. All assessments were operated by a fixed and trained researcher to ensure error reduction.

#### 2.4.3. FMD test.

FMD of the brachial artery diameter was using an instrument equipped with software for monitoring the brachial artery (UNEX38G, Japan), and the measurements were taken in 20 to 25 minutes. All volunteers were lying in supine and relaxed position with a blood pressure cuff placed around their left forearm. The brachial artery was scanned longitudinally 5 to 10 cm above the elbow by an ultrasonic echo. When the clearest image of the anterior and posterior intimal interfaces between the lumen and vessel wall was obtained, both tracking gates were placed on the intima, the artery diameter was automatically tracked, and the waveform of diameter changes over a cardiac cycle was displayed in real time using the FMD mode of the tracking system. After measuring the blood pressure, the blood pressure cuff was inflated to 50 mm Hg above the systolic pressure for 5 minutes and released. Blood vessel diameter changes were measured for 2 minutes after the 5 minutes inflation. Measurement of the minimum blood vessel diameter of the baseline diameter was defined for 20 seconds after releasing the blood pressure cuff. FMD was automatically calculated as a percentage change in peak vessel diameter from the baseline value. The reference value was 7% or more, and endothelial dysfunction was suspected in <4%. The whole measured process was completed by the professional operator.

#### 2.4.4. HRV analysis.

About the HRV collection, the volunteers were instructed to have a 10 minutes supine resting before the test, and measured using the SphygmoCor device (AtCor Medical, Australia) with beat-to-beat records using RR intervals. The time series records for HRV analysis, we collected the standard deviation of normal-to-normal intervals recorded in 1 time interval and square root of the mean squared differences of successive normal-to-normal intervals; frequency domain indexes analyzed using spectral analysis: low-frequency spectral component expressed in normalized units (LF) (with a variation of 0.04–0.15 Hz, expressed in ms) and LF n.u.: LF; high frequency spectral components, high frequency spectral component expressed in normalized units (HF) (with a variation of 0.15 to 0.4 Hz, expressed in ms) and HF n.u.: HF; cardiac sympathovagal balance assessed by means of the ratio between low and high frequency components; the total power spectral area comprising the entire spectrum of frequencies, up to the maximum limit of 0.50 Hz, which expresses the global autonomic nervous system function. All measured process was completed by the trained operator.

#### 2.4.5. Circulating biomarkers analysis.

Blood samples were collected after an 8-hour fast at the first visit, between 7:00 to 11:00. All blood draws were performed at the Scientific Research Center at the Guangzhou Sport University at Guangzhou. On the visit, 10 mL of venous blood was collected and centrifuged for 10 minutes (Centribio 80-2B, Brazil) for serum separation, and then stored in a refrigerator (BioFreezer, Germany) at − 80 °C for analysis. Total cholesterol (TC), high-density lipoprotein (HDL), low-density lipoprotein, triglycerides, fasting glucose and fasting insulin were analyzed, blood samples were tested using a Hitachi 747 autoanalyzer (Hitachi, Japan). Arterial stiffness index (AI) was calculated as the [TC (mmol/L)- HDL (mmol/L)]/ HDL (mmol/L). Matrix metalloproteinase-2 (MMP-2), matrix metalloproteinase-9 (MMP-9), norepinephrine (NA), neuropeptide Y (NP-Y), interleukin-6 (IL-6), tumor necrosis factor-α (TNF-α), C-reactive protein (CRP), peptide Y (PY-Y), ghrelin, leptin, and adiponectin were analyzed using an Enzyme-linked immunosorbent assay according to manufacturer’s instructions (Meimian Biotechnology, China).

### 2.5. Statistical analysis

All analyses were performed using SPSS 25.0 (IBM SPSS Amos, USA). Data of characteristics of participants are showed as mean ± standard deviation for continues variables or as n (%) for categorical variables. The association of cardiovascular risk (arterial stiffness, autonomic nervous system function, and biomarkers) with lifestyle habits (sedentary behaviors, physical activity, sleep habits and eating habits) were evaluated by Pearson Bivariate Correlation analysis, and correlation coefficient (r) was used to estimate the strength and direction of the association, where *R* ≤ 0.30 was considered as small, 0.30 < *R* < 0.80 as moderate, and *R* ≥ 0.80 as large. A 2-tailed *P* value of <.05 is used to indicate statistical significance. Individuals with missing data were excluded from the analysis.

## 3. Results

### 3.1. Characteristics of participants

The anthropometric, circulating metabolic, sedentary behaviors, and physical activity data are summarized in Table 1. A total of 60 sedentary adults (35 % females) were analyzed in the present study, with a mean age of 23.63 ± 3.74 years (18 to 35 years). Mean values for waist–hip ratio was 0.90 ± 0.08, that for fat mass was 27.86 ± 9.85%, and body mass index was 26.90 ± 5.97 kg/m2. The circulating lipid biomarkers including TC (4.74 ± 1.24 mmol/L), triglycerides (1.63 ± 2.61 mmol/L), and low-density lipoprotein (2.76 ± 1.00 mmol/L) but except for HDL (0.81 ± 0.26 mmol/L), were in normal range; glucose metabolic markers including fasting glucose (4.63 ± 0.55 mmol/L) and fasting insulin (14.03 ± 13.72 uIU/mL) were within normal range. Accelerometer measured the average value of maximal length of sedentary bout of the included participants was 57.72 ± 5.45 minutes/week; the average value of total sedentary break was 8469.53 ± 530.10 minutes/week; the average value of total time in sedentary behavior was 6407.63 ± 748.29 minutes/week (15.26 ± 1.78 hours/day). Accelerometer measured moderate to vigorous physical activity time was 947.03 ± 291.04 minutes/week (2.25 ± 0.69 hours/day) and total time in physical activity was 2579.40 ± 541.68 minutes/week (6.14 ± 1.29 hours/day).

**Table 1:** Characteristics of participants (n = 60).

Variables	All
Age (yrs)	23.63 (3.74)
Male	39 (65)
Waist–hip ratio	0.90 (0.08)
Fat mass (%)	27.86 (9.85)
BMI (kg/m²)	26.90 (5.97)
Metabolic biomarkers	
Total cholesterol (mmol/L)	4.74 (1.24)
Triglycerides (mmol/L)	1.63 (2.61)
High-density lipoproteins (mmol/L)	0.81 (0.26)
Low-density lipoproteins (mmol/L)	2.76 (1.00)
Fasting glucose (mmol/L)	4.63 (0.55)
Fasting insulin (uIU/mL)	14.03 (13.72)
Sedentary behaviors	
Maximum length of sedentary bouts (min/wk)	54.72 (5.45)
Total length of sedentary breaks (min/wk)	8469.53 (530.10)
Total time in sedentary behavior (min/wk)	6407.63 (748.29)
Physical activity	
Moderate to vigorous physical activity time (min/wk)	947.03 (291.04)
Total time in physical activity (min/wk)	2579.40 (541.68)

Continuous variables are presented as mean (SD); categorical variables are presented as frequency (%).

BMI = body mass index.

### 3.2. Correlation between lifestyle habits and arterial stiffness

#### 3.2.1. Correlation between sedentary behaviors/physical activity and arterial stiffness.

As shown in Table 2, there seemed to be no statistical association between sedentary behaviors and most function parameters for arterial stiffness except for total time in sedentary behavior with ankle-brachial index left (r = −0.313, *P* = .015); similar results were observed between physical activity and all function parameters. As for circulating biomarkers indicating arterial stiffness, maximum length of sedentary bouts, total length of sedentary breaks, and total time in physical activity were significantly associated with MMP-2 (*P* < .05) and MMP-9 (*P* < .05).

**Table 2:** Correlation between arterial stiffness and lifestyle habits among sedentary adults (n = 60).

Variables	Function parameters						Circulating biomarkers		
	Bapwv L (m/s)	Bapwv R (m/s)	Cfpwv (m/s)	ABI L	ABI R	FMD (%)	AI	MMP-2 (ng/mL)	MMP-9 (ug/L)
Sedentary behaviors									
Maximum length of sedentary bouts (min/wk)	−0.183 (0.163)	−0.248 (0.056)	−0.226 (0.082)	−0.313 (0.015)*	−0.138 (0.292)	0.078 (0.553)	0.213 (0.101)	−0.284 (0.028)*	−0.272 (0.035)*
Total length of sedentary breaks (min/wk)	0.040 (0.761)	0.190 (0.147)	0.090 (0.495)	0.071 (0.592)	0.005 (0.971)	−0.175 (0.181)	−0.020 (0.879)	0.278 (0.031)*	0.267 (0.039)*
Total time in sedentary behavior (min/wk)	0.110 (0.403)	−0.008 (0.954)	0.104 (0.429)	−0.109 (0.408)	−0.003 (0.980)	0.165 (0.208)	0.139 (0.289)	−0.083 (0.530)	−0.083 (0.528)
Physical activity									
Moderate to vigorous physical activity time (min/wk)	−0.094 (0.474)	−0.081 (0.539)	−0.117 (0.372)	0.153 (0.244)	0.119 (0.127)	−0.231 (0.075)	−0.089 (0.497)	0.172 (0.188)	0.182 (0.164)
Total time in physical activity (min/wk)	−0.066 (0.615)	−0.036 (0.787)	−0.072 (0.587)	0.033 (0.805)	0.085 (0.518)	−0.167 (0.203)	−0.115 (0.383)	0.294 (0.022)*	0.301 (0.019)*
Sleep habits									
Subjective sleep quality	−0.279 (0.031)*	−0.269 (0.038)*	−0.312 (0.015)*	0.081 (0.537)	−0.046 (0.729)	0.204 (0.858)	0.144 (0.271)	−0.031 (0.816)	0.016 (0.904)
Sleep duration	0.075 (0.570)	−0.031 (0.816)	0.026 (0.844)	0.253 (0.051)	0.182 (0.165)	0.051 (0.700)	0.268 (0.038)*	−0.092 (0.483)	−0.084 (0.522)
Habitual sleep efficiency	0.044 (0.737)	−0.077 (0.560)	−0.003 (0.984)	0.161 (0.219)	0.124 (0.134)	−0.113 (0.389)	0.045 (0.735)	0.234 (0.072)	0.259 (0.045)*
Use of sleep medication	−0.241 (0.063)	−0.316 (0.014)*	−0.269 (0.038)*	−0.054 (0.679)	0.109 (0.408)	0.004 (0.977)	0.042 (0.750)	−0.018 (0.892)	−0.026 (0.846)
PSQI	−0.011 (0.933)	−0.073 (0.580)	−0.024 (0.857)	−0.009 (0.946)	−0.061 (0.644)	0.049 (0.709)	0.360 (0.005)*	−0.048 (0.716)	−0.019 (0.886)
Sleep efficiency (%)	−0.260 (0.045)*	−0.266 (0.040)*	−0.298 (0.021)*	−0.107 (0.418)	−0.325 (0.011)*	−0.180 (0.169)	0.176 (0.179)	−0.412 (0.001)*	−0.381 (0.003)*
Average total sleep time (min/d)	−0.297 (0.021)*	−0.272 (0.036)*	−0.310 (0.016)*	0.084 (0.526)	−0.074 (0.575)	−0.233 (0.073)	−0.037 (0.780)	−0.131 (0.317)	−0.123 (0.350)
Wake after sleep onset (min)	0.015 (0.907)	0.067 (0.612)	0.073 (0.580)	0.129 (0.328)	0.323 (0.012)*	0.081 (0.537)	−0.232 (0.074)	0.335 (0.009)*	0.302 (0.019)*
Eating habits									
Food responsiveness	0.000 (0.997)	0.101 (0.441)	0.028 (0.830)	−0.016 (0.906)	0.047 (0.724)	0.125 (0.342)	−0.085 (0.518)	−0.139 (0.290)	−0.132 (0.315)
Satiety responsiveness	−0.115 (0.382)	−0.045 (0.730)	−0.085 (0.517)	0.141 (0.282)	0.005 (0.970)	−0.216 (0.097)	−0.210 (0.108)	0.110 (0.402)	0.076 (0.563)
Emotional overeating	0.276 (0.033)*	0.263 (0.042)*	0.265 (0.041)*	0.205 (0.116)	−0.096 (0.468)	0.058 (0.662)	−0.244 (0.060)	0.139 (0.288)	0.154 (0.239)
Emotional under-eating	−0.045 (0.730)	−0.080 (0.545)	−0.055 (0.675)	−0.096 (0.466)	0.128 (0.331)	0.062 (0.636)	0.106 (0.418)	−0.152 (0.247)	−0.146 (0.266)
Enjoyment of food	0.002 (0.987)	−0.082 (0.535)	−0.057 (0.665)	−0.226 (0.082)	−0.196 (0.133)	0.283 (0.028)*	0.033 (0.802)	−0.145 (0.268)	−0.139 (0.290)
Food fussiness	0.082 (0.535)	0.250 (0.055)	0.165 (0.209)	0.044 (0.736)	0.080 (0.543)	−0.178 (0.173)	0.058 (0.660)	−0.004 (0.974)	0.007 (0.960)
Slowness in eating	−0.019 (0.885)	0.061 (0.642)	−0.001 (0.991)	0.077 (0.556)	−0.019 (0.884)	−0.196 (0.134)	−0.273 (0.035)*	−0.045 (0.733)	−0.051 (0.700)

Data were analyzed using Pearson Bivariate Correlation; values are r (*P* value).

ABI L = ankle-brachial index left, ABI R = ankle-brachial index right, AI = atherosclerosis index, Bapwv L = brachial-ankle pulse wave velocity left, Bapwv R = brachial-ankle pulse wave velocity right, Cfpwv = carotid-femoral pulse wave velocity, FMD = flow mediated dilation, MMP-2 = matrix metalloproteinase-2, MMP-9 = matrix metalloproteinase-9, PSQI = pittsburgh sleep quality index.

* indicates *P* < .05.

#### 3.2.2. Correlation between sleep habits and arterial stiffness.

Results based on questionnaire of sleep habits showed that both subjective sleep quality (*P* < .05) and use of sleep medication (*P* < .05) were inversely associated with function parameters of arterial stiffness with significant differences, which included brachial-ankle pulse wave velocity right (Bapwv R) and Cfpwv. In addition, sleep habits derived from accelerometer were closely associated with those function parameters. For example, sleep efficiency was significantly associated with brachial-ankle pulse wave velocity left (Bapwv L) (r = −0.260, *P* = .045), Bapwv R (r = −0.266, *P* = .040), and Cfpwv (r = −0.298, *P* = .021); average total sleep time was also significantly associated with Bapwv L (r = −0.297, *P* = .021), Bapwv R (r = −0.272, *P* = .036), and Cfpwv (r = −0.310, *P* = .016). As for circulating biomarkers indicating arterial stiffness, average total sleep time and wake after sleep onset were significantly associated with MMP-2 (*P* < .05) and MMP-9 (*P* < .05).

#### 3.2.3. Correlation between eating habits and arterial stiffness.

The questionnaire results of eating habits seem had no relation with most function parameters of arterial stiffness except for emotional overeating with Bapwv L (*R* = 0.276, *P* = .033), Bapwv R (*R* = 0.263, *P* = .042) and Cfpwv (*R* = 0.265, *P* = .041), and enjoyment of food with FMD (*R* = 0.283, *P* = .028). Similarly, the circulating biomarkers indicating arterial stiffness were not associated with eating habits except for slowness in eating with AI (r = −0.273, *P* = .035).

### 3.3. Correlation between lifestyle habits and autonomic nervous system function

#### 3.3.1. Correlation between sedentary behaviors/physical activity and autonomic nervous system function.

As shown in Table 3, no statistical association between sedentary behaviors and most function parameters for autonomic nervous system function was observed except for total time in sedentary behaviors with root mean square of successive RR interval differences (r = −0.269, *P* = .038) and total power spectral area (r = −0.292, *P* = .023); similar results were observed between physical activity and all function parameters. With regard to circulating biomarkers of autonomic nervous system function, only total length of sedentary breaks was significantly associated with NP-Y (*R* = 0.255, *P* = .050), and there had statistical association between total time in physical activity and NA (*R* = 0.312, *P* = .015) and NP-Y (*R* = 0.269, *P* = .037).

**Table 3:** Correlation between autonomic nervous system and lifestyle habits among sedentary adults (n = 60).

Variables	Function parameters						Circulating biomarkers	
	RMSSD (ms)	SDNN (ms)	LF (n.u.)	HF (n.u.)	LF/HF (ms²)	TP (ms)	NA (ng/L)	NP-Y (ng/L)
Sedentary behaviors								
Maximum length of sedentary bouts (min/wk)	−0.040 (0.763)	0.186 (0.155)	0.126 (0.338)	−0.126 (0.338)	0.080 (0.543)	−0.053 (0.688)	−0.214 (0.100)	−0.248 (0.056)
Total length of sedentary breaks (min/wk)	−0.007 (0.955)	−0.171 (0.192)	0.169 (0.197)	−0.169 (0.197)	0.093 (0.478)	0.042 (0.752)	0.246 (0.058)	0.255 (0.050)*
Total time in sedentary behavior (min/wk)	−0.269 (0.038)*	−0.063 (0.633)	0.144 (0.273)	−0.144 (0.273)	0.232 (0.074)	−0.292 (0.023)*	−0.138 (0.292)	−0.091 (0.491)
Physical activity								
Moderate to vigorous physical activity time (min/wk)	−0.056 (0.671)	0.110 (0.404)	0.136 (0.300)	−0.136 (0.300)	0.004 (0.977)	−0.006 (0.964)	0.185 (0.158)	0.145 (0.269)
Total time in physical activity (min/wk)	−0.093 (0.481)	0.098 (0.457)	0.247 (0.057)	−0.247 (0.057)	0.063 (0.632)	0.035 (0.793)	0.312 (0.015)*	0.269 (0.037)*
Sleep habits								
Subjective sleep quality	0.021 (0.872)	−0.040 (0.760)	−0.005 (0.968)	0.005 (0.968)	0.042 (0.749)	−0.129 (0.326)	0.051 (0.697)	0.019 (0.883)
Sleep duration	−0.063 (0.632)	−0.046 (0.730)	−0.031 (0.815)	0.031 (0.815)	−0.126 (0.336)	0.016 (0.906)	−0.066 (0.618)	−0.059 (0.656)
Habitual sleep efficiency	−0.165 (0.209)	−0.023 (0.859)	0.055 (0.678)	−0.055 (0.678)	−0.069 (0.600)	−0.053 (0.686)	0.266 (0.040)*	0.262 (0.043)*
Use of sleep medication	−0.081 (0.540)	−0.039 (0.769)	−0.041 (0.756)	0.041 (0.756)	−0.058 (0.659)	−0.004 (0.974)	0.001 (0.995)	0.016 (0.901)
PSQI	−0.084 (0.523)	0.053 (0.689)	0.115 (0.383)	−0.115 (0.380)	0.211 (0.106)	−0.117 (0.375)	−0.011 (0.935)	−0.013 (0.922)
Sleep efficiency (%)	−0.086 (0.516)	−0.142 (0.279)	−0.204 (0.117)	0.204 (0.117)	−0.119 (0.366)	0.039 (0.768)	−0.283 (0.028)*	−0.357 (0.005)*
Average total sleep time (min/d)	0.089 (0.498)	−0.154 (0.239)	−0.268 (0.038)*	0.268 (0.038)*	−0.250 (0.054)	−0.188 (0.150)	−0.075 (0.572)	−0.078 (0.554)
Wake after sleep onset (min)	0.155 (0.236)	0.160 (0.222)	0.038 (0.772)	−0.038 (0.772)	−0.002 (0.989)	−0.095 (0.471)	0.210 (0.108)	0.290 (0.025)*
Eating habits								
Food responsiveness	0.032 (0.809)	0.040 (0.763)	−0.155 (0.237)	0.155 (0.237)	0.037 (0.780)	−0.007 (0.956)	−0.146 (0.266)	−0.150 (0.251)
Satiety responsiveness	0.060 (0.649)	−0.011 (0.934)	−0.283 (0.028)*	0.283 (0.028)*	−0.364 (0.004)*	0.093 (0.479)	0.063 (0.633)	0.053 (0.688)
Emotional overeating	−0.172 (0.190)	0.217 (0.096)	−0.159 (0.226)	0.159 (0.226)	−0.062 (0.636)	−0.109 (0.409)	0.164 (0.210)	0.151 (0.250)
Emotional under-eating	−0.016 (0.905)	0.088 (0.503)	0.039 (0.765)	−0.039 (0.765)	−0.052 (0.695)	−0.062 (0.638)	−0.216 (0.097)	−0.200 (0.125)
Enjoyment of food	0.000 (0.999)	−0.023 (0.863)	0.306 (0.017)*	−0.306 (0.017)*	0.371 (0.004)*	0.163 (0.214)	−0.155 (0.237)	−0.159 (0.225)
Food fussiness	0.145 (0.268)	0.218 (0.094)	−0.125 (0.342)	0.125 (0.342)	−0.131 (0.317)	−0.095 (0.472)	0.037 (0.780)	−0.022 (0.870)
Slowness in eating	0.017 (0.898)	−0.026 (0.844)	−0.242 (0.062)	0.242 (0.062)	−0.298 (0.021)*	−0.027 (0.841)	−0.050 (0.702)	−0.045 (0.732)

Data were analyzed using Pearson Bivariate Correlation; values are r (*P* value).

HF = high frequency spectral component expressed in normalized units, LF = low-frequency spectral component expressed in normalized units, LF/HF = ratio between low and high frequency components, NA = norepinephrine, NP-Y = neuropeptide Y, PSQI = pittsburgh sleep quality index, RMSSD = root mean square of successive RR interval differences, SDNN = standard deviation of normal-to-normal intervals recorded in one time interval, TP = total power spectral area.

* indicates *P* < .05.

#### 3.3.2. Correlation between sleep habits and autonomic nervous system function.

Based on the questionnaire results of sleep habits, no significant relationship was observed in all function parameters of autonomic nervous system function. Furthermore, accelerometer measured sleep habits had no relation with most function parameters of autonomic nervous system function except for average total sleep time with LF (r = −0.268, *P* = .038) and HF (*R* = 0.268, *P* = .038). As regards circulating biomarkers of autonomic nervous system function, questionnaire of habitual sleep efficiency and accelerometer measured sleep efficiency were significantly associated with NA (*P* < .05) and NP-Y (*P* < .05).

#### 3.3.3. Correlation between eating habits and autonomic nervous system function.

Results from questionnaire of eating habits showed that both satiety responsiveness (*P* < .05) and enjoyment of food (*P* < .05) were inversely associated with function parameters of autonomic nervous system function with significant differences, including LF, HF, and ratio between low and high frequency components. No statistical association between eating habits and circulating biomarkers indicating autonomic nervous system function was observed in the current results.

### 3.4. Correlation between lifestyle habits and circulating biomarkers

As shown in Table 4, maximum length of sedentary bouts was significantly associated with CRP (*P* < .05), PY-Y (*P* < .05), ghrelin (*P* < .05), and leptin (r = −0.294, *P* = .023), and significant associations were also observed with both total length of sedentary breaks and total time in physical activity.

**Table 4:** Correlation between lifestyle habits and circulating biomarkers among sedentary adults (n = 60).

Variables	Inflammatory biomarkers			Appetite-regulating biomarkers		Adipokines	
	IL-6 (ng/L)	TNF-α (ng/L)	CRP (ug/L)	PY-Y (pg/mL)	Ghrelin (ng/L)	Leptin (ug/L)	Adiponectin (pg/mL)
Sedentary behaviors							
Maximum length of sedentary bouts (min/wk)	−0.173 (0.187)	−0.228 (0.080)	−0.294 (0.023)*	−0.264 (0.041)*	−0.309 (0.016)*	−0.294 (0.023)*	−0.017 (0.900)
Total length of sedentary breaks (min/wk)	0.230 (0.077)	0.237 (0.069)	0.321 (0.012)*	0.325 (0.011)*	0.301 (0.019)*	0.306 (0.017)*	0.021 (0.874)
Total time in sedentary behavior (min/wk)	−0.081 (0.539)	−0.091 (0.488)	−0.059 (0.654)	−0.051 (0.700)	−0.072 (0.586)	−0.078 (0.552)	−0.068 (0.604)
Physical activity							
Moderate to vigorous physical activity time (min/wk)	0.018 (0.894)	0.104 (0.431)	0.188 (0.151)	0.158 (0.227)	0.201 (0.124)	0.159 (0.224)	0.007 (0.956)
Total time in physical activity (min/wk)	0.183 (0.161)	0.248 (0.056)	0.328 (0.011)*	0.264 (0.042)*	0.316 (0.014)*	0.282 (0.029)*	0.067 (0.613)
Sleep habits							
Subjective sleep quality	0.006 (0.964)	−0.004 (0.976)	0.003 (0.980)	−0.101 (0.443)	−0.064 (0.625)	−0.056 (0.673)	−0.032 (0.810)
Sleep duration	−0.010 (0.940)	−0.021 (0.876)	−0.101 (0.442)	−0.019 (0.887)	−0.111 (0.399)	−0.099 (0.451)	−0.282 (0.029)*
Habitual sleep efficiency	0.255 (0.049)*	0.285 (0.027)*	0.252 (0.052)	0.309 (0.016)*	0.218 (0.094)	0.206 (0.113)	0.008 (0.952)
Use of sleep medication	0.052 (0.693)	0.025 (0.852)	−0.068 (0.607)	−0.021 (0.872)	−0.053 (0.686)	−0.014 (0.914)	−0.160 (0.222)
PSQI	−0.004 (0.979)	−0.016 (0.901)	−0.029 (0.829)	−0.058 (0.662)	−0.065 (0.621)	−0.068 (0.604)	−0.140 (0.287)
Sleep efficiency (%)	−0.289 (0.025)*	−0.270 (0.037)*	−0.261 (0.044)*	−0.387 (0.002)*	−0.409 (0.001)*	−0.407 (0.001)*	−0.044 (0.739)
Average total sleep time (min/d)	−0.012 (0.928)	−0.029 (0.826)	−0.105 (0.423)	−0.087 (0.509)	−0.161 (0.218)	−0.108 (0.412)	0.106 (0.418)
Wake after sleep onset (min)	0.223 (0.086)	0.206 (0.114)	0.187 (0.153)	0.334 (0.009)*	0.318 (0.013)*	0.337 (0.009)*	0.132 (0.313)
Eating habits							
Food responsiveness	−0.148 (0.258)	−0.170 (0.194)	−0.088 (0.503)	−0.140 (0.288)	−0.126 (0.339)	−0.133 (0.310)	0.030 (0.821)
Satiety responsiveness	0.109 (0.405)	0.052 (0.694)	0.086 (0.514)	0.060 (0.648)	0.073 (0.577)	0.075 (0.568)	0.381 (0.003)*
Emotional overeating	0.125 (0.340)	0.109 (0.408)	0.069 (0.600)	0.080 (0.544)	0.110 (0.401)	0.110 (0.404)	−0.072 (0.585)
Emotional under-eating	−0.190 (0.146)	−0.249 (0.055)	−0.128 (0.329)	−0.182 (0.164)	−0.149 (0.256)	−0.176 (0.178)	0.187 (0.152)
Enjoyment of food	−0.182 (0.165)	−0.201 (0.123)	−0.138 (0.294)	−0.191 (0.144)	−0.143 (0.275)	−0.158 (0.228)	0.021 (0.873)
Food fussiness	−0.076 (0.564)	−0.070 (0.596)	0.005 (0.971)	−0.016 (0.903)	−0.019 (0.887)	−0.004 (0.977)	−0.077 (0.561)
Slowness in eating	−0.023 (0.860)	−0.034 (0.795)	−0.098 (0.458)	−0.039 (0.767)	−0.075 (0.570)	−0.065 (0.619)	0.193 (0.139)

Data were analyzed using Pearson Bivariate Correlation; values are r (*P* value).

CRP = C-reactive protein, IL-6 = interleukin-6, PSQI = pittsburgh sleep quality index, PY-Y = peptide YY, TNF-α = tumor necrosis factor-α.

* indicates *P* < .05.

The questionnaire results of sleep habits showed that sleep duration was negatively associated with adiponectin (r = −0.282, *P* = .029), but habitual sleep efficiency was positively associated with IL-6 (*R* = 0.255, *P* = .049), TNF-α (*R* = 0.285, *P* = .027), and PY-Y (*R* = 0.309, *P* = .016). Moreover, accelerometer measured sleep efficiency were significantly associated with circulating biomarkers, such as IL-6 (r = −0.289, *P* = .025), TNF-α (r = −0.270, *P* = .037), CRP (r = −0.261, *P* = .044), PY-Y (r = −0.387, *P* = .002), ghrelin (r = −0.409, *P* = .001), and leptin (r = −0.407, *P* = .001); wake after sleep onset was positively associated with PY-Y (*R* = 0.334, *P* = .009), ghrelin (*R* = 0.318, *P* = .013), and leptin (*R* = 0.337, *P* = .009). Besides, there seemed to be no statistical association between eating habits and most circulating biomarkers except for satiety responsiveness with adiponectin (*R* = 0.381, *P* = .003).

## 4. Discussion

The current study examined the relationship of lifestyle habits (including physical activity, sleep habits, and eating habits) with cardiovascular risk represented by arterial stiffness and autonomic nervous system function among sedentary adults. The results showed that: Total time in physical activity was positively associated with the circulating levels of both MMPs and sympathetic markers; Healthy sleep habits were closely associated with functions and circulating biomarkers indicating arterial stiffness, and also with high level of circulating sympathetic markers; Unhealthy eating habits (emotional overeating) was positively associated with arterial stiffening, and other eating habits (including satiety responsiveness, enjoyment of food, and slowness in eating) were notably associated with sympathetic activation; Total time in physical activity and sedentary behaviors, and sleep efficiency were significantly associated with circulating biomarkers. These finding highlighted the critical importance of keeping healthy lifestyle habits, including sufficient physical activity and good sleep and eating habits, among sedentary adults on decreasing cardiovascular risk.

In this study, breaking prolong sedentariness (represented by maximum length of sedentary bouts and total length of sedentary breaks) was significantly associated with circulating levels of MMPs, and total time spent on sitting was negatively associated with parasympathetic activation; notably, total time in physical activity was significantly associated with those circulating biomarkers indicating both arterial stiffness (MMPs) autonomic nervous system function (NA, NP-Y), inflammatory biomarker (CRP), appetite-regulating biomarkers (PY-Y and ghrelin), and adipokine (leptin). Consistently, previous studies supported that adults who spent longer duration of sedentary behaviors are more likely to present higher level of arterial stiffness and dysfunction of autonomic nervous system function. For example, quantitative evidence from randomized trials^[27]^ concluded great improvements of FMD after both short-and long-term sedentary behaviors interventions among adults. As for autonomic nervous system function, longer time spent on sitting is linked with poor cardiac autonomic modulation^[28]^ and is even the main lifestyle-related factor associated with this autonomic regulation^[29]^; in addition, HRV (the balance between sympathetic and parasympathetic nervous system) was significantly decreased by even shorter term intervention of sedentary behaviors.^[30]^ Physical activity, as an independent lifestyle habit but also strongly linked with sedentary behaviors, has been generally accepted to be associated with lower arterial stiffness, and any intensity of physical activity is favorable to improve PWV in adults.^[31]^ Moreover, total time spent on physical activity is associated with higher activity of parasympathetic modulation and lower of sympathetic modulation, improving cardiac autonomic modulation,^[32]^ and the recent evidence demonstrated that 6-week of high intensity physical activity could effectively ameliorate autonomic imbalance among insufficiently active adults.^[33]^ Indeed, data from meta-analysis concluded that exercise training increases cardiac parasympathetic nervous modulation among sedentary people.^[34]^

In addition to more physical activity and less sedentariness, high quality of sleep was another key lifestyle factor responsible for the reduced cardiovascular risk. Therefore, our data showed that significant associations were found of healthy sleep habits (represented by subjective sleep quality, use of sleep medication, sleep efficiency, and average total sleep time) with lower PWV parameters, and of bad sleep efficiency (sleep efficiency and wake after sleep onset) with ankle-brachial index right, inflammatory biomarkers (IL-6, TNF-α, and CRP), appetite-regulating biomarkers (PY-Y and ghrelin), and leptin. Evidence from large population samples also supported that long sleep duration was associated with higher PWV,^[35]^ and sleep could even moderate the association between arterial stiffness and blood pressure variability.^[36]^ Those strong associations were confirmed by levels of circulating biomarkers in the current study. In addition, average total sleep time was negatively associated with sympathetic modulation and positively associated with parasympathetic activation; healthy sleep habits (represented by sleep efficiency and wake after sleep onset) were significantly associated with circulating biomarkers indicating sympathetic activity (NA and NP-Y). Consistently, prior studies supported that health sleep habits were linked with lower arterial stiffness and improved cardiac autonomic modulation,^[37]^ and our study further highlighted the importance of enough sleep time and good sleep efficiency on decreasing cardiovascular risk among sedentary adults.

Unhealthy eating habits were also a key lifestyle factor responsible to increase cardiovascular risk among sedentary adults. In this study, correlation was found of emotional overeating with PWV parameters, of enjoyment of food with FMD, and of slowness in eating with circulating levels of AI. As for autonomic nervous system function, satiety responsiveness, enjoyment of food, and slowness in eating were significantly associated with the balance of sympathetic and parasympathetic nervous system. Those eating behaviors based on questionnaire suggested diet is a modifiable lifestyle factor and would make a substantial contribution to the risk of cardiovascular among sedentary adults. Other evidence derived from food frequency questionnaire also showed that compliance with health diet recommendations was related to lower PWV and lower cardiovascular risk.^[38]^ In addition, emotional overeating was significantly associated with autonomic nervous system activity,^[39]^ available evidence showed that dysfunction of autonomic nervous system might also lead to emotional eating habits.^[40]^ Interventional studies were encouraged to infer the causal relationship between eating habits and autonomic nervous function. Collectively, unhealthy eating habits jointly with sedentary behaviors would increase cardiovascular risk among adults.

## 5. Limitations

This study has several limitations. Firstly, due to the cross-sectional design of this study, the correlative nature of the data precludes us from establishing a causal relationship. Secondly, other unhealthy lifestyle habits were not included in this study, such as high alcohol intake, smoking, and medicine abuse. Despite these limitations, we believe that our findings provide basic evidence for the association between lifestyle habits (determined by multiple factors) and cardiovascular risk (evaluations of both function parameters and circulating biomarkers) among sedentary adults. Future interventional and large population studies are needed in order to evaluate further relationships of lifestyle habits with cardiovascular risk.

## 6. Conclusion

This study verified that lifestyle habits represented by physical activity, sleep habits, and eating habits were closely associated with arterial stiffness and autonomic nervous system regulation, which indicate cardiovascular risk among sedentary adults. For people with prolonging sitting time due to work or habits, more time on physical activity and efficient sleep, and avoiding emotional overeating should be encouraged to decrease arterial stiffness and to balance autonomic nervous function.

## Author contributions

**Conceptualization:** Min Hu, Jingwen Liao.

**Formal analysis:** Linyu Peng, Jingwen Liao.

**Funding acquisition:** Shen Wang, Min Hu, Jingwen Liao.

**Investigation:** Linyu Peng, Lidan Chen, Shen Wang, Lianmeng Guo, Wenhao Liang, Jie Zhou, Niujin Shi.

**Methodology:** Linyu Peng, Lidan Chen.

**Project administration:** Min Hu, Jingwen Liao.

**Resources:** Linyu Peng, Shen Wang, Jingwen Liao.

**Software:** Linyu Peng, Lidan Chen.

**Supervision:** Linyu Peng, Jingwen Liao.

**Validation:** Linyu Peng.

**Visualization:** Linyu Peng.

**Writing – original draft:** Linyu Peng, Jingwen Liao.

**Writing – review & editing:** Linyu Peng, Junhao Huang, Min Hu.
